# The Synergistic Effect of Three Essential Oils against Bacteria Responsible for the Development of Lithiasis Infection: An Optimization by the Mixture Design

**DOI:** 10.1155/2021/1305264

**Published:** 2021-08-28

**Authors:** Rabie Kachkoul, Ghita Benjelloun Touimi, Bahia Bennani, Radouane El Habbani, Ghita El Mouhri, Mohamed Mohim, Tarik Sqalli Houssaini, Mohamed Chebaibi, Amine Koulou, Anissa Lahrichi

**Affiliations:** ^1^Laboratory of Biochemistry, Faculty of Medicine and Pharmacy, University Sidi Mohammed Ben Abdellah, BP 1893, Km 22, Road of Sidi Harazem, Fez, Morocco; ^2^Faculty of Science and Technology, University Sidi Mohammed Ben Abdellah, BP 2202, Road of Imouzzer, Fez, Morocco; ^3^Laboratory of Human Pathology Biomedicine and Environment, Faculty of Medicine and Pharmacy of Fez, Sidi Mohammed Ben Abdellah University (USMBA), Fez, Morocco; ^4^Laboratory of Molecular Bases in Human Pathology and Therapeutic Tools, Faculty of Medicine and Pharmacy, University Sidi Mohammed Ben Abdellah, BP 1893, Km 22, Road of Sidi Harazem, Fez, Morocco; ^5^Department of Nephrology, University Hospital Hassan II, BP 1835, Atlas, Road of Sidi Harazem, Fez, Morocco; ^6^Biomedical and Translational Medical Research Center, Faculty of Medicine and Pharmacy of the Fez, University of Sidi Mohamed Ben Abdellah, Fez 30000, Morocco; ^7^Laboratory of Catalyse Organic Chemistry and Environment, Faculty of Sciences, Ibn Tofail University, Kenitra, Morocco

## Abstract

The present study aimed to determine the chemical composition and the synergistic effect of three plants' essential oils (EOs), *Eucalyptus camaldulensis* (ECEO), *Mentha pulegium* (MPEO), and *Rosmarinus officinalis* (ROEO), against three bacterial strains, *Proteus mirabilis*, *Klebsiella pneumoniae*, and *Staphylococcus aureus*, in order to increase the antimicrobial effectiveness by the use of a low dose of essential oils, consequently decreasing the toxicity and negative impact. For this reason, an augmented simplex-centroid mixture design was used to build polynomial models in order to highlight the synergy between the essential oils against bacterial strains. Antimicrobial effect screening was performed by the disc diffusion method and the minimal inhibitory concentrations (MIC) were also studied. The gas chromatography/mass spectrometry (GC-MS) results show the richness of these essential oils by terpenic compounds, especially 1,8-Cineole and P-Cymene for ECEO, Pulegone for MPEO, and *α*-Pinene and Camphene for ROEO. Moreover, a significant antibacterial effect has been demonstrated and the best values were revealed by MPEO and ECEO against *P. mirabilis* and *K. pneumoniae,* with inhibition zones (IZ) of 25 and 20 mm, respectively, and an MIC of 0.0391% (v:v) against *K. pneumoniae*. The optimal mixtures showed a synergistic effect of essential oils, and the lowest minimal inhibitory concentrations of the mixtures (MIC_m_) were in the order of 29.38% of MPEO, 45.37% of ECEO, and 25.25% of ROEO against *P. mirabilis* and in the order of 60.61% of MPEO and 39.39% of ROEO against *K. pneumoniae*. These results indicate the antibacterial efficacy of the three essential oils combined and suggest their importance in the treatment of urinary tract infections caused by resistant bacterial strains.

## 1. Introduction

Urinary tract infection is one of the most common bacterial infections in women and men, affecting more than 150 million people around the world every year [1–3]. It can cause life-threatening septicemia, but most infections are less severe [4]. However, this infection is a risk factor for lithogenesis, which can be the cause of infectious stones, especially carbapatite and struvite. These stones may also be secondary to a nonurinary infectious agent, *Oxalobacter formigenes*, as well as nanobacteria [5].

Infection stones are formed as part of an infection of the upper urinary tract by urease-producing bacteria (*Proteus, Klebsiella pneumoniae*, *Pseudomonas aeruginosa*, *Staphylococcus aureus*, *Aspergillus fumigatus*, and *Enterobacter*) [6, 7]. These microorganisms hydrolyze urea to produce ammonia and hydroxide, increasing the urine pH and therefore increasing the dissociation of phosphate to form trivalent phosphate. The latter bind with magnesium to form a “triple crystal” of struvite (magnesium ammonium phosphate) and/or calcium carbonate apatite stones. Those stones generally develop in branched form (staghorn), which occupies a large part of the collector system [8, 9].

In revanche, essential oils have gained increased interest and are considered as an alternative for the fight against bacterial infections, especially drug resistance [10–13]. Furthermore, the individual compounds of the plants often act in synergy so as to potentiate the activity of the combination significantly compared to that of the individual components [14].

*Eucalyptus* is a genus native to Australia, which belongs to the Myrtaceae family [15], represented by more than 700 species distributed around the world [16–18]. This genus is known to be a rich source of bioactive compounds, such as phenolic acids, flavonoids, and hydrolyzable tannins, as well as essential oils [15, 19–24]. However, many biological activities were mentioned: antioxidant, antibacterial, antifungal, insecticide, antiviral, antiseptic, antituberculosis, and antiquorum detection activities [18, 24–27].

*Mentha pulegium* L. (Fleyou») is an aromatic, herbaceous perennial plant that belongs to the Lamiaceae family and reaches up to 40 cm in height [28, 29]. The plant is endemic in Europe, North Africa, Asia Minor, and the Middle East [29, 30]. It is known for its carminative, antispasmodic, antiseptic, diaphoretic and emmenagogue analgesic, diuretic, antihypertensive, anti-inflammatory, antioxidant, antimicrobial, and insecticide activities. It is also used for the treatment of fevers, headaches, minor respiratory infections, digestive disorders, menstrual disorders, and various minor ailments [28–32]. The chemical composition of the plant is rich in volatile compounds such as Pulegone, Isomenthone, 1,8-Cineole, Piperitone, and Piperitenone [28, 33–35].

*Rosmarinus officinalis* (Rosemary) is a perennial culinary plant that belongs to the Lamiaceae family, originally from the Mediterranean region [36–38]. Rosemary has an amalgam of biological activities including anti-inflammatory, antioxidant, antibacterial, hepatoprotective, antithrombotic, diuretic, antidiabetic, antinociceptive, anticancer, antimutagenic, antidepressant, antiatherosclerotic, and antiparasitic activity, and it is used as a chemopreventive and antispasmodic agent to reduce rheumatism, improve digestion, and relieve stomach pain [37–44]. In addition, the plant's chemical composition is rich in essential oils, terpenoids, flavonoids, and phenolic acids [43–48].

In this regard, this study was focused on determining the antibacterial effects of the combined essential oils of three plants, *Eucalyptus camaldulensis*, *Mentha pulegium*, and *Rosmarinus Officinalis*, against three bacterial strains: *P. mirabilis*, *K. pneumoniae*, and *S. aureus*. This combination was chosen in order to increase the efficiency and minimize the dose of essential oils, thus decreasing their toxicity and negative impact. For this, a simplex-centroid augmented mixture design was used to build polynomial models in order to highlight the synergy between the essential oils against bacterial strains. In addition, the chemical composition of EOs has been identified and the correlation between these compounds and the antibacterial activity has also been determined.

## 2. Materials and Methods

### 2.1. Essential Oil Extraction

Samples of *Mentha pulegium* and *Eucalyptus camaldulensis* were collected in the Taounate region (located in the north of Morocco, 92 km from Fez city, 34°32′09 ″N, 4°38′24″ W). The sample of *Rosmarinus officinalis* was harvested at the Botanical Garden of the Faculty of Medicine and Pharmacy-Fez. Taxonomic identification was performed by Professor A. Bari and Botanical voucher specimens have been deposited in the Laboratory of Biotechnology, Environment, Agri-Food and Health Herbarium (Faculty of Sciences Dhar El-Mahraz, Sidi Mohammed Ben Abdellah University, Fez, Morocco) under the following references: 137-2021T1, 137-2021 T, and 137-2021 FMPF.

Essential oils from the *Eucalyptus camaldulensis* leaves and the aerial parts of *Mentha pulegium* and *Rosmarinus Officinalis* were extracted by the hydrodistillation method using the Clevenger-type equipment. 100 g of each sample was subjected to hydrodistillation for 4 hours at the water boiling temperature (100°C). Once extracted, the essential oils obtained were dried on anhydrous sodium sulfate, stored at a temperature of 4°C in dark glass flacons until use [49].

### 2.2. Gas Chromatography/Mass Spectrometry (GC-MS) Analysis of Essential Oils

The essential oils have been analyzed on a Thermo Fischer Trace GC ULTRA gas chromatograph coupled to a mass spectrometer (Polaris Q MS with ion trap). The gas chromatography device is equipped with a VB-5 (Methylpolysiloxane 5% phenyl) column (30 m^*∗*^0.25 mm^*∗*^0.25 *µ*m). The gas used is Helium with a flow rate of 1.4 mL/min and samples are injected in split mode. The injection temperature and injected volume are 220°C and 1 *µ*L, respectively. The column initial temperature is 40°C for 2 min and it increases from 40°C to 180°C at a rate of 4°C·min^−1^ and from 180°C to 300°C at a rate of 20°C·min^−1^, and the final temperature is maintained for 2 min. The mass spectrometer operates under the following conditions: fragmentation is carried out by electronic impact under a field of 70 eV; the source and the interface are maintained at 200 and 300°C. The mass spectra are recorded over an m/z range from 50 to 650 with 0.5 s/scan. The identification of each separate chemical compound is carried out on the basis of its mass spectra compared to those in the NIST database.

### 2.3. Antibacterial Activity

#### 2.3.1. Bacterial Strains

The bacterial strains used in this work were obtained from the Microbiology and Molecular Biology Laboratory of the Faculty of Medicine and Pharmacy, University Sidi Mohammed Ben Abdellah, Fez, Morocco. The *P. mirabilis* and *K. pneumoniae* strains are *Bacillus* Gram-negative, while the *S. Aureus* strain is *Cocci* Gram-positive.

#### 2.3.2. Antibiogramme

The susceptibility and resistance of bacteria to antibiotics were determined by the disc diffusion method on Mueller-Hinton (MH) agar. The antibiogram was performed in accordance with the standardization criteria defined by the Antibiogram Committee of the Microbiology French Society, 2019 edition [50]. The antibiotics used are Erythromycin (E) 15 *µ*g, Ampicillin (AMP) 10 *µ*g, Ceftazidime (CAZ) 10 *µ*g, Oxacillin (OX) 1 *µ*g, Ofloxacin (OFX) 5 *µ*g, Ticarcillin (TIC) 75 *µ*g, Norfloxacin (NOR) 10 *µ*g, and Cefotaxime (CTX) 5 *µ*g.

#### 2.3.3. Inoculum Preparation

From a bacterial culture (24 hours), identical colonies were scraped off using a sealed Pasteur pipette. A volume of 10 mL was discharged into a sterile saline solution (0.9%), the bacterial suspension was homogenized, and its opacity was reduced to 0.5 McFarland corresponding to 10^7^ CFU·mL^−1^. After that, the suspension was diluted to give an inoculum of 10^6^ CFU·mL^−1^ [51].

#### 2.3.4. Disc Diffusion Method

The Agar diffusion method allows predicting with certainty the in vitro efficacy of the essential oils and the antibiotics; it is in fact a qualitative assessment of the activity. It was carried out by the protocol described by Abdelli et al. [51] with some modifications.

Each strain is subcultured into 2 ml of Mueller-Hinton broth solution (BMH) and incubated at 37°C for 2.5 to 3 hours. Subsequently, 20 ml of Mueller-Hinton agar medium is poured into a Petri dish, and once the agar has cooled, the bacterial inoculum is inoculated by the swabbing technique. After 5 min, a sterile filter paper disc with a diameter of 6 mm is aseptically deposited on the surface of each plate and 10 *μ*l essential oil is added. In parallel, a virgin witness in essential oil is prepared. The Petri dishes are left for 1 hour at 4°C and then inverted and incubated at 37°C for 18 to 24 hours. After incubation, the inhibition diameter is measured in mm, including the disc [51].

#### 2.3.5. Minimum Inhibitory Concentration (MIC) Determination

The broth microdilution method was used to evaluate the MIC, using the dimethyl sulfoxide (DMSO) as an emulsifier and triphenyl tetrazolium chloride (TTC) as an indicator of bacterial growth. 20 *µ*l of DMSO was distributed from the second to the twelfth well of the 96-well microplate (Greiner, VWR). Later, 40 *µ*l of the essential oil was added to the first test well of each line in the microplate, from which 20 *µ*L geometric base 2 dilution was made from the second to the eleventh well. The twelfth well was considered a growth control. Then, 160 *μ*L of Mueller-Hinton Broth (BMH) and 20 *μ*L of a 10^6^ CFU·ml^−1^ bacterial suspension are added to all wells. After 18 hours of incubation at 37°C, the reading was taken by adding 10 *µ*L of color indicator (TTC) diluted in sterile distilled water in the order of 0.2 g·ml^−1^, followed by incubation for 10 min at 37°C. The TTC reveals the presence of live bacteria by the appearance of red coloration [13, 52].

#### 2.3.6. Antibacterial Effect of a Three-Essential-Oils Mixture by Mixture Design

The mixture designs are a specific branch of the experimental designs. The response in this plan depends only on the relative proportions of the factors and not on the quantities of mixture used, which must be between zero and one and their sum equal to one (or 100%). Lower and upper limits may be imposed (for one or more factors) for security reasons or due to economic constraints [53–55].

This experimental design methodology was used to find the optimal formulation while minimizing the experiments number. Thus, it allows determining the relationship between the variables and the experimental responses measured. In our study, the optimization aimed at finding the constituents of the formulation giving the best essential oils combination allowing the highest antibacterial activity, which is illustrated by minimum inhibitory concentration of the mixtures MIC_m_.

#### 2.3.7. Experimental Matrix and Mathematical Model

**Figure 1:**
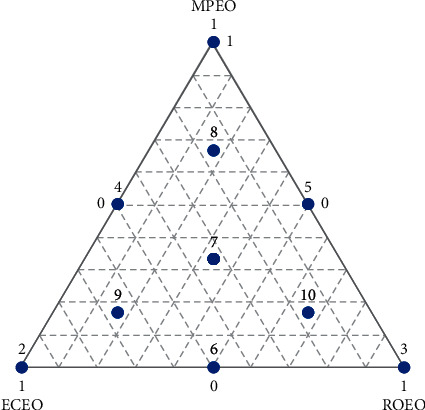
Plot of the augmented simplex-centroid design. ECEO: *Eucalyptus camaldulensis* essential oil; MPEO: *Mentha pulegium* essential oil; ROEO: *Rosmarinus officinalis* essential oil.

The simplex-centroid augmented design was chosen to optimize and determine the synergistic antibacterial effect of the three essential oils: ECEO, MPEO, and ROEO. This design includes ten experiments distributed as follows: the three EOs in the triangle's vertices (experiments 1, 2, and 3), the binary mixtures 0.5/0.5 (experiments 4, 5, and 6), the mixture in equal proportions of the three constituents (experiments 7), and control points (experiments 8, 9, and 10) (Figure 1). Experiment 7 was replicated three times to determine the pure error and compare it with the lack of fit. Consequently, the number of experiments for this design was equal to 12 (Table 1) [12, 13].

The responses were the antibacterial effects of EOs quantified as minimum inhibitory concentration of the mixtures MIC_m_ and were evaluated by the microdilution method. Then, the data were fitted to a special cubic polynomial model using least-squares regression to estimate the unknown coefficients in the following equation:

(1) $$\begin{array}{c} Y\;=\;b1X1\;+\;b2X2\;+\;b3X3\;+\;b12X1X2\;+\;b13X1X3\;+\;b23X2X3\;+\;b123X1X2X3, \end{array}$$

where *Y* is the overall response of the mixture. *X*1, *X*2, and *X*3 are the proportions of the components in the mixture. *b*1, *b*2, and *b*3 are the magnitudes of the effect from each component. *b*12, *b*13, and *b*23 are the magnitudes of the interaction effect of two components. *b*123 is the magnitude of the interaction effect of the three components.

#### 2.3.8. Minimum Inhibitory Concentration of the Mixtures (MIC_m_)

The MIC_m_ of the three EOs mixtures were carried out in the same way as in Section 2.3.5 with the change in concentration of the stock solution. In this work, we used the concentrations that gave the MIC of each EO against each bacterial strain as the stock solution in order to see if there are any agonist or antagonist interactions between its EOs and to avoid the over-effect of one EO on the other EOs.

#### 2.3.9. Statistical Analysis

Test design and statistical analysis for model validation were performed using Minitab 18 software. The ratio between the mean square due to regression (CMR) and the residual mean square (CMr), *F* ratio (R/r), was used at a significance level of 95% to check the statistical significance of the model. The variability of the data around its mean is explained adequately by the higher *F* value. The quality of the first-order polynomial fit was also expressed by the coefficient of determination *R*^2^. This coefficient measures the adequacy of the regression equation (model) with the experimental data. In fact, it measures the correlation between observed and predicted responses and is often expressed as a percentage. Student's *t*-test was used at a significance level of 95% to confirm or reject the significance of the factors. In the table of coefficients, each factor is associated with the values of Student's *t*-test and *p* value. Student's *t*-test values are used to determine the significance of the regression coefficients for each parameter and the *p* values are defined as the lowest level of importance leading to the rejection of the null hypothesis [13]. The principal component analysis (PCA) was carried out using IBM SPSS Statistics 20 software.

## 3. Results

### 3.1. Essential Oils' Chemical Composition

The identification of the chemical compounds in each EO was based on the comparison of their mass spectra with those of the NIST database. Indeed, the results of the ECEO, MPEO, and ROEO chemical compounds identification are represented in Tables 2–4 .

The chemical composition analysis of *Eucalyptus camaldulensis* leaves' essential oil revealed 67 compounds representing 96.48% of the total oil (Table 2). In fact, the terpene composition consists mainly of monoterpenes, with 45.92% oxygenated monoterpenes and 25.08% hydrocarbon monoterpenes. Meanwhile, sesquiterpenes represent only 12.3% of those oxygenated and 8.07% of the hydrocarbons. The main compounds of this oil are 1,8-Cineole (19.05%), P-Cymene (17.06%), (−)-Spathulenol (9.42%), Cryptone (5.99%), Phellandral (5.34%), and Cuminaldehyde (4.56%). Our chromatographic profile is almost in concordance with another study by Elgat et al. [56]. However, Medhi et al. [18] found a higher proportion of 1,8-Cineole (69.46%) followed by *γ*-Terpinene (15.10%). Meanwhile, Knezevic et al. [57] reported a variation in the chemical compound proportion of this EO between samples collected from two different geographical areas. The proportion of P-Cymene found in this work is greater than that of Farah et al. [58] which are worked on the samples harvested from the experimental plot EU. PL25 (Sidi Yahia du Gharb, Northwest of Morocco) and its natural hybrid are collected from the experimental plot Ell. II (forest zone of Sidi Slimane du Gharb, Northwest of Morocco). Nevertheless, other compounds such as 1,8-Cineole and *α*-Pinene are presented with significant proportion, especially in hybrid samples [58, 59].

The GC-MS analysis result of the *Mentha pulegium* aerial part essential oil shown in Table 3 displays the presence of 45 compounds, regrouping a cumulative area corresponding to 95.77% of the total constituents. Oxygenated monoterpenes represent the majority of terpene with a percentage of 77.09% against 5.27 and 3.69% for hydrocarbon monoterpenes and oxygenated sesquiterpenes, respectively. In addition, Pulegone is the predominant compound with a rate of 51.02%, followed by Isopulegone (6.69%), Piperitenone (5.90%), and (−)-1R-8-Hydroxy-p-menth-4-en-3-one (4.08%). The same majority compound was found in the works of Brahmi et al. [60], Abdelli et al. [51], Bouyahya et al. [61], and Chraibi et al. [62]. The two latter works have been accomplished by the Moroccan samples.

50 chemical compounds that represent 97.78% of the total accumulated air were identified in *Rosmarinus officinalis* EO (Table 4). This latter is marked by the abundance of hydrocarbon monoterpenes (60.96%), followed by oxygenated monoterpenes (28.16%) and hydrocarbon sesquiterpenes (5.58%). In addition, *α*-Pinene (24.90%), Camphene (9.32%), D-Limonene (7.15%), (+)-Camphor (6.11%), and *α*-Fenchene (4.24%) are the majority compounds. However, the results obtained for this oil are close to those of Liu et al. [63] who reported *α*-Pinene as the majority compound. The study conducted by Ainane et al. [64] revealed the dominance of the (−)-Camphor compound. Moreover, 1,8-Cineole was identified as the majority compound in the works of Capatina et al. [65], Barreto et al. [66], and Selmi et al. [67].

### 3.2. Agar Disc Diffusion-Screening of the Antibacterial Effect of the Essential Oils and Resistance to Antibiotics

The qualitative demonstration of the antibacterial effect of essential oils (ECEO, MPEO, and ROEO) and antibiotics on three bacterial strains, *P. mirabilis*, *K. pneumoniae*, and *S. aureus*, was evaluated by the disk diffusion method; the results of the inhibition zones are shown in Table 5.

The antibiotic effects screening indicates that the strains studied in this work have a very high resistance profile against antibiotics, hence its importance to find alternatives to these agents. However, according to Table 5, *K. pneumoniae* is sensitive to three antibiotics (OFX, NOR, and CTX) among the seven evaluated. *S. aureus* is sensitive to OFX and NOR, while *P. mirabilis* is resistant to all antibiotics. Moreover, the inhibition zones for pure essential oils (Table 5) show that the MPEO revealed a very important antimicrobial effect against the three strains, with values of 20, 20, and 10 mm, while the ECEO values are 25, 12, and 10 mm for *P. mirabilis*, *K. pneumoniae*, and *S. aureus*, respectively. Meanwhile, the ROEO profile marks no effect on the *S. aureus* strain.

### 3.3. Minimum Inhibitory Concentration (MIC)

The results of the minimum inhibitory concentration (MIC) of the three plant essential oils are shown in Table 6.

According to Table 6, all the essential oils studied display a significant MIC, except for ROEO which shows no reaction against *S. aureus* (Gram-positive). The recorded concentrations are with respect to 0.3125, 0.0391, and 0.0781% (v:v) for ECEO and 0.3125, 0.0391, and 0.625% (v:v) for PMEO against *P. mirabilis*, *K. pneumoniae* (Gram-negative), and *S. aureus* (Gram-positive), respectively. Meanwhile ROEO reveals a concentration of 2.5 and 10% (v:v) against the first two strains.

### 3.4. Principal Component Analysis (PCA)

**Figure 2:**
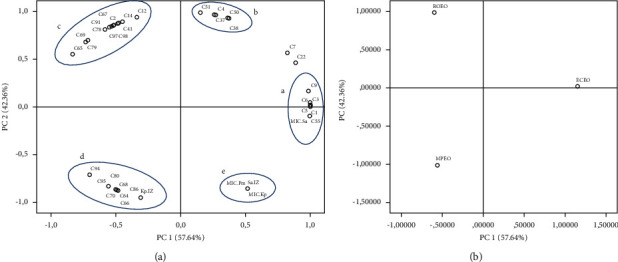
Principal component analysis. (a) Loading plot of chemical compounds and antibacterial activity. (b) Score plot of the principal components and EOs samples distribution. ECEO: *Eucalyptus camaldulensis* essential oil; MPEO: *Mentha pulegium* essential oil; ROEO: *Rosmarinus officinalis* essential oil. Kp.IZ: *K. pneumoniae* inhibition zone; Sa.IZ: *S. aureus* inhibition zone; MIC.Kp: minimum inhibitory concentration against *K. pneumoniae*; MIC.Pm: minimum inhibitory concentration against *P. mirabilis*; MIC.Sa: minimum inhibitory concentration against *S. aureus.* Chemical compounds' abbreviations (C) are presented in Tables 2–4.

**Figure 3:**
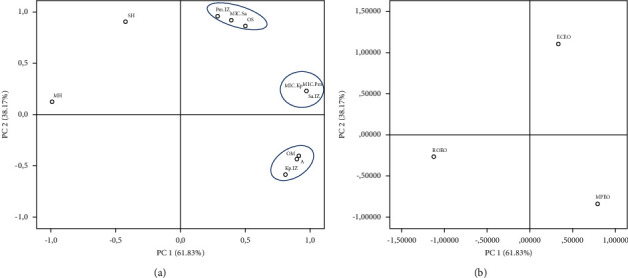
Principal component analysis. (a) Loading plot of EOs chemicals classes and antibacterial activity. (b) Score plot of the principal components and EOs samples distribution. ECEO: *Eucalyptus camaldulensis* essential oil; MPEO: *Mentha pulegium* essential oil; ROEO: *Rosmarinus officinalis* essential oil. Kp.IZ: *K. pneumoniae* inhibition zone; Pm.IZ: *P. mirabilis* inhibition zone; Sa.IZ: *S. aureus* inhibition zone; MIC.Kp: minimum inhibitory concentration against *K. pneumoniae*; MIC.Pm: minimum inhibitory concentration against *P. mirabilis*; MIC.Sa: minimum inhibitory concentration against *S. aureus.* Chemicals classes abbreviation are presented in Tables 2–4.

The principal component analysis was applied to highlight the relationship between the EOs chemical composition of three plants studied and their antibacterial activities. In fact, the results are shown in Figures 2 and 3 .

According to Figure 2(a), the first component (PC1) represents 57.64% of the total variation and is dominated mainly by hydrocarbon monoterpenes (*α*-Thujene, *β*-Myrcene, Thuja-2, 4(10)-diene, and (−)-*β*-Pinene) and MIC against *S. aureus* (group a). Meanwhile, the second component (PC2) represents 42.36% of the variability and is linked principally to hydrocarbon sesquiterpenes (*β*-Cadinene, *γ*-Muurolene, and *α*-Copaene) (group b). The loading plot shows also three other groups; group c includes hydrocarbon and oxygenated monoterpenes (*β*-Pinene, *α*-Fenchene, D-Limonene, *α*-Pinene, *γ*-Terpinene, Linalool, *α*-Terpineol, Verbenone, 3-Carene, and Cyclofenchene) and a hydrocarbon sesquiterpene (*β*-Caryophyllene); these variables are correlated with each other and have a weak positive correlation with PC2 and a negative one with PC1.

The variables gathered in group d are also the hydrocarbon and oxygenated monoterpenes principally (O-Cymene, p-Mentha-3, 8-diene, Pulegone, and Piperitenone), the oxygenated sesquiterpenes (Caryophyllene oxide and Humulene epoxide II), the compound 3-Octanol, and the growth inhibition zone of *K. pneumoniae* variable, but this group is anticorrelated with the two PCs. Group e regrouped the variables that are related to the antimicrobial activity against the three bacterial strains studied.

The score plot (Figure 2(b)) explores the correlations between the PCs and the studied essential oils, making it possible to determine which variables discriminate these three EOs. Indeed, ECEO has a strong score for PC1 which is linked to group a variables, and groups c and d discriminate ROEO and MPEO, respectively.

The PCA which compares the chemical class proportion and the antibacterial activity of the three studied EOs plants (Figure 3) reveals a correlation between the *P. mirabilis* inhibition zone, MIC against *S. aureus*, and the oxygenated sesquiterpene; these variables characterize the ECEO. Meanwhile MPEC is characterized by a high proportion of oxygenated monoterpene and an inhibitory effect against the *K. pneumoniae* bacterial strain growth. This result may explain the important effect of oxygenated terpene compounds in inhibiting bacterial growth.

### 3.5. Optimization of the Antibacterial Effect of a Three-Essential-Oils Mixture by the Mixture Design

The optimization of the essential oils mixture's antibacterial effect against three bacterial strains, *P. mirabilis*, *K. pneumoniae*, and *S. aureus*, has been determined by MIC_m_. Recalling that, in this section, the concentrations of the stock solutions were selected from the MICs found in Section 3.3 for each bacterial strain, indeed, the observed responses for each experiment are displayed in Table 7.

### 3.6. Statistical Validation of the Model Postulated

Statistical analysis of the experimental response data corresponding to each bacterial strain was carried out in order to verify the special cubic model chosen, which describes the relationship between factors and responses. The results of the findings are shown in Table 8.

According to Table 8, the analysis of variance (ANOVA) shows that the *F* ratio (R/r) calculated for *P. mirabilis* (9.733) and *K. pneumoniae* (6.178) is higher than the tabular value (4.95) at the 95% confidence level. In addition, the *p* value is in order of 0.0123 and 0.0321 (<0.05), respectively. In fact, these results prove that the regression main effect is statistically significant for these two models. Moreover, the coefficients of determination *R*^2^ for *P. mirabilis* and *K. pneumoniae* are 0.92 and 0.88, respectively, which is an indicator of the correlation between the experimental and predicted values in the adapted mathematical model. However, the regression main effect is statistically insignificant for the model that examines the responses of *S. aureus* with an *F* ratio (R/r) of 2.349 and a *p* value of 0.183, and the coefficient of determination *R*^2^ = 0.74 displays the insufficiency of the correlation; therefore the model will be excluded.

### 3.7. Effect of the Mixture Components, Their Interactions, and the Models Applied

The interaction between different essential oil compounds can reduce or increase antimicrobial efficiency. These interactions can produce four types of results: indifferent, additive, antagonistic, and synergistic results [12]. However, the effects of all factors studied, the statistical values of Student's *t*-test, and *p* value are reported in Table 9.

The interpretation of the models data relating the responses to the factors (Table 9) shows that the coefficients of the terms that represent the effects of the pure components (*b*1, *b*2, and *b*3) are significant against the *P. mirabilis* and *K. pneumoniae* bacterial strains, with *p* value values less than 0.05. Binary interactions between MPEO and ECEO (b12) and between ECEO and ROEO (*b*23) are significant against *P. mirabilis* (*p* < 0.05), and interactions between MPEO and ROEO (*b*13) and between ECEO and ROEO (*b*23) are significant against *K. pneumoniae* (*p* < 0.05). Meanwhile the coefficients of the ternary interaction terms are not significant (*p* > 0.05) and show no effect on the two bacterial strains. In fact, after eliminating all nonsignificant coefficients from the postulated models, the mathematical models representing the response in terms of the three components are represented by equations (2) and (3) for *P. mirabilis* and *K. pneumoniae*, respectively.

(2) $$\begin{array}{c} Y\;=\;9.40\mathrm{MPEO}+10.08\mathrm{ECEO}+4.86\mathrm{ROEO}-31.02\mathrm{MPEO}{}^{*}\mathrm{ECEO}-20.11\mathrm{ECEO}{}^{*}\mathrm{ROEO,} \end{array}$$

(3) $$\begin{array}{c} Y\;=\;5.25\mathrm{MPEO}+\;9.57\mathrm{ECEO}+\;9.57\mathrm{ROEO}-20.35\mathrm{MPEO}{}^{*}\mathrm{ROEO}-21.71\mathrm{ECEO}{}^{*}\mathrm{ROEO}. \end{array}$$

**Figure 4:**
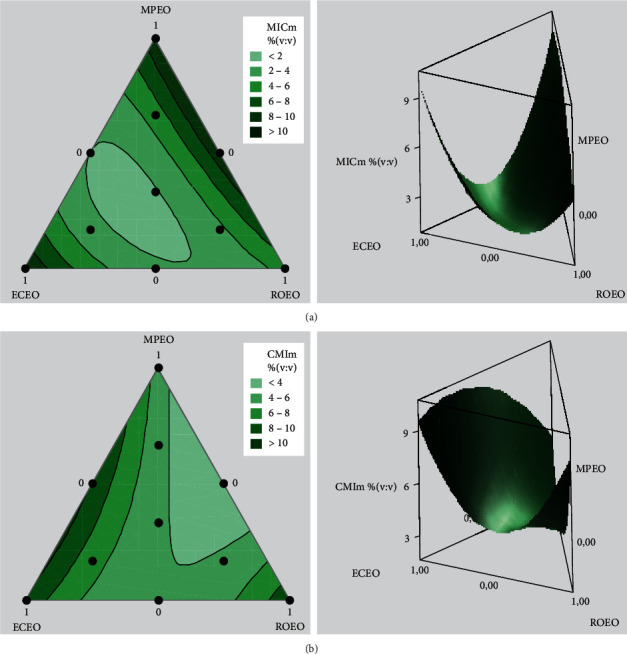
2D contour and 3D surface responses (CMI_m_) graphs against *P.mirabilis* (a) and *K. pneumoniae* (b). ECEO: *Eucalyptus camaldulensis* essential oil; MPEO: *Mentha pulegium* essential oil; ROEO: *Rosmarinus officinalis* essential oil; MIC_m_: minimum inhibitory concentration of the mixtures.

In general, coefficients with positive signs for mixtures indicate that the two components act synergistically or are complementary, resulting in an increased response. Meanwhile, negative coefficients suggest an antagonistic effect relative to each other; therefore there is a decrease in response. In fact, this study aims to minimize the response that represents MIC_m_ values; hence the coefficient with a negative sign reflects the ability of its associated factor to increase the antibacterial effect. However, the binary combination of MPEO and ECEO exhibits a significant synergistic effect against *P. mirabilis*. A significant synergistic effect against *K. pneumoniae* was revealed by the combination of MPEO and ROEO. Meanwhile the interaction between ECEO and ROEO has a significant synergistic effect against these two bacterial strains. These results are clearly observed in the 2D contour and 3D surface plots in Figure 4.

### 3.8. Mixture Optimization

**Figure 5:**
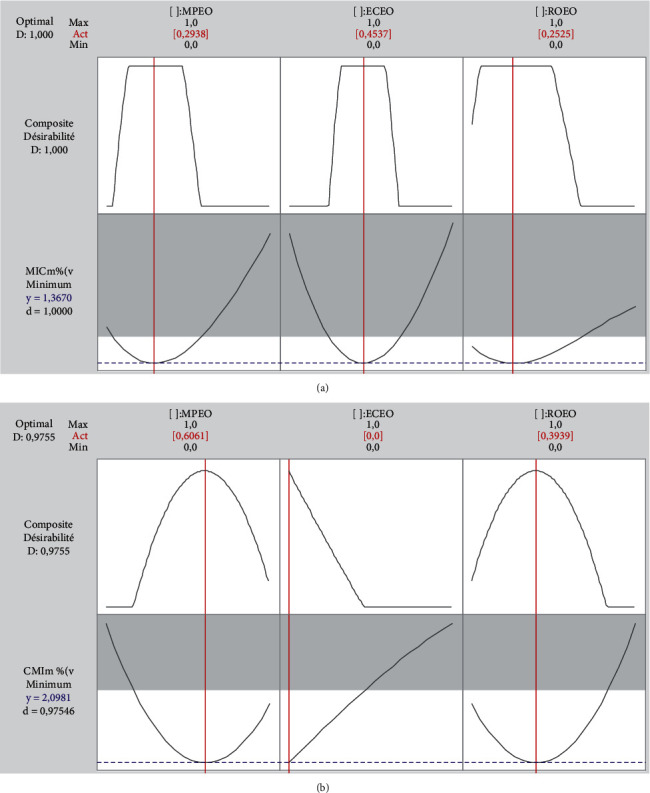
Desirability profiles of optimal essential oil proportions relative to a lowest MIC for *P.mirabilis* (a) and *K. pneumoniae* (b). ECEO: *Eucalyptus camaldulensis* essential oil; MPEO: *Mentha pulegium* essential oil; ROEO: *Rosmarinus officinalis* essential oil; MIC_m_: minimum inhibitory concentration of the mixtures.

Mixture optimization was evaluated by the desirability function method in order to obtain a formulation of the optimal essential oil proportions resulting in a lowest MIC_m_. The results obtained are illustrated in Figure 5.

According to the desirability profile of antimicrobial activity against *P. mirabilis* (Figure 5(a)), the lowest MIC_m_ value can reach 1.367% with desirability of 100%. This value can be obtained by mixing essential oils with proportions of 29.38% for MPEO, 45.37% for ECEO, and 25.25% for ROEO. Regarding *K. pneumoniae* (Figure 5(b)), a mixture of 60.61% MPEO and 39.39% ROEO predicts an MIC_m_ of 2,098%, with 97,55% desirability. This result confirms the hypothesis of synergy in the binary combination between MPEO and ROEO.

Moreover, the optimal essential oils concentrations in the mixture generating a minimal MIC_m_ against bacterial strains were calculated by the following equation:

(4) $$\begin{array}{c} \text{optimal essential oil concentration}=\text{MICm}{}^{*}\text{DF of the initial EO concentration}{}^{*}\%\text{ essential oil found by the mixing design}. \end{array}$$

DF is the dilution factor; MIC_m_ is the minimum inhibitory concentration of the mixtures.

The minimum concentrations found against *P. mirabilis* are around 0.0126% (v:v) for MPEO, 0.0194% (v:v) for ECEO, and 0.0863% (v:v) for ROEO. A mixture with a concentration of 0.0050% (v:v) for MPEO and 0.8264% (v:v) for ROEO is able to inhibit *K. pneumoniae* growth.

## 4. Discussion

This study demonstrates the potential of *Eucalyptus camaldulensis, Mentha pulegium*, and *Rosmarinus officinalis* essential oils and their combination against three bacterial strains, *P. mirabilis*, *K. pneumoniae*, and *S. aureus*, which are marked by antibiotic resistance. The inhibition zones revealed by the disc diffusion method (Table 5) prove the individual efficacy of these EOs, especially by MPEO, while the RMEO shows no reaction against *S. aureus*. In fact, the inhibition zones for the latter reported by Bozin et al. [47] and Safaei-Ghomi and Ahd [25] with essential oils of *Rosmarinus officinalis* and eucalyptus, respectively, were superior to ours, but this strain studied in their work does not mark antibiotics-resistance (Penicillin and Gentamicin); similarly, Mattazi et al. [68] have found an antibacterial effect against *S. aureus* and *Klebsiella* by *Rosmarinus officinalis* samples collected from the Biougra region (province of Chtouka Ait Baha, Agadir city. Morocco). Furthermore, our results of the MPEO effect against *P. mirabilis* are similar to those reported by Abdelhakim et al. who worked on the *Mentha pulegium* plant from the Ouezzane region (Northwest Morocco) [61]. In addition, the minimum inhibitory concentration (MIC) method, which allows determining the lowest EOs concentration that is able to inhibit the bacteria growth, confirms the screening results found by the disc diffusion method.

The data obtained related to the mixture EOs optimization show the importance of binary combinations in increasing the antibacterial effect, as well as the decrease of these essential oils' concentrations and consequently the reduction of toxicity. However, the GC-MS analysis of the EOs studied in this work showed their richness in bioactive chemical compounds, the PCA revealed the correlations between these latter and the antibacterial effect, and consequently these compounds probably act individually or in synergy on bacterial strains. In addition, Ložienė et al. [69] have established a very strong antibacterial effect of the *α*-Pinene fractions with an enantiomers mixture (1S)-(−) < (1R)-(+) *α*-Pinene as well as the (1R)-(+)-*α*-Pinene standard against *S. aureus* ATCC, with an MIC of 0.01% (w:v). Similarly, Pulegone [70], 1,8-Cineole, Camphore, Verbenone, and Borneol [71], and Menthol and Menthone [72] have also shown a great capacity to inhibit this last bacterial strain. The study performed by Vuuren and Viljoen [73] reveals a remarkable antimicrobial activity of 1,8-Cineole compound against the *K. pneumoniae* strain with an MIC of 8 mg/ml. Meanwhile Shahverdi et al. [74] reported that the antimicrobial activity of Furazolidone and Nitrofurantoin (a marketed antibacterial agent) against *K. pneumoniae* and *Proteus* spp. increases with the presence of Piperitone.

However, the essential oil mechanisms action remains less clear, and their complexity comes from the diversity of chemical molecules, each of which can act on a different target [75, 76]. Several EOs antibacterial mechanisms have been described by Bouyahya et al. [76]; these action mechanisms include cell membrane crossing, potassium leakage and respiratory chain disruption, impairment of cell division, and quorum detection signalling pathways inhibition resulting in decreased bacterial resistance [61, 76, 77].

In conclusion, this work highlighted the chemical composition and the antimicrobial efficacy of *Mentha pulegium*, *Eucalyptus camaldulensis*, and *Rosmarinus officinalis* plants' essential oils, as well as their combinations against three bacterial strains, *P. mirabilis*, *K. pneumoniae*, and *S. aureus*. The analysis by GC-MS shows the richness of ECEO by 1,8-Cineole and P-Cymene, and MPEO has a very high level of Pulegone, while *α*-Pinene and Camphene are the major components of ROEO. The disc diffusion method has demonstrated the effectiveness of these essential oils in growth inhibition of the first two bacterial strains and especially MPEO. Meanwhile *S. aureus* shows resistance against ROEO. These results were confirmed by the MIC method. The optimization of the combination between the three essential oils by the mixture design shows the validity of the models that examine the responses of MIC_m_ against *P. mirabilis* and *K. pneumoniae*. The synergistic effect between essential oils has been demonstrated, and the optimal mixtures that reveal the lowest MIC_m_ values against these last bacterial strains are in order of 29.38% MPEO, 45.37% ECEO, and 25.25% ROEO against *P. mirabilis* and in order of 60.61% MPEO and 39.39% ROEO against *K. pneumoniae*. These results suggest that these essential oils can be used as antimicrobial agents, especially against resistant bacterial strains.

## Figures and Tables

**Table 1:** Different combinations of the essential oils contents chosen by the mixture design.

Experiment	MPEO (*X*1)	ECEO (*X*2)	ROEO (*X*3)
1	1.00	0.00	0.00
2	0.00	1.00	0.00
3	0.00	0.00	1.00
4	0.50	0.50	0.00
5	0.50	0.00	0.50
6	0.00	0.50	0.50
7	0.33	0.33	0.33
8	0.67	0.17	0.17
9	0.17	0.67	0.17
10	0.17	0.17	0.67
11	0.33	0.33	0.33
12	0.33	0.33	0.33

ECEO: *Eucalyptus camaldulensis* essential oil; MPEO: *Mentha pulegium* essential oil; ROEO: *Rosmarinus officinalis* essential oil.

**Table 2:** Chemical composition of *Eucalyptus camaldulensis* essential oil.

No.	Compound	Abbreviation	RT	Area (%)
1	*α*-Thujene	C1	11.12	1.48
2	*α*-Pinene	C2	11.29	2.57
3	Thuja-2,4(10)-diene	C3	11.46	0.46
4	*β*-Terpinene	C4	11.98	0.17
5	(−)-*β*-Pinene	C5	12.1	0.2
6	*β*-Myrcene	C6	12.18	0.65
7	*α*-Phellandrene	C7	12.53	1.23
8	*α*-Terpinene	C8	12.74	0.43
9	P-Cymene	C9	12.97	17.06
10	1,8-Cineole	C10	13.1	19.05
11	Dehydrolinalool	C11	13.27	0.73
12	*γ*-Terpinene	C12	13.42	0.71
13	3.5-Dimethylstyrene	C13	13.93	0.87
14	Linalool	C14	13.99	0.37
15	Inconnu	C123	14.04	0.24
16	*α*-Thujone	C15	14.42	0.31
17	(+)-trans-4-Thujanol	C16	14.49	0.45
18	*α*-Campholenal	C17	14.56	0.12
19	cis-(+/−)-4-Thujanol	C18	14.77	0.21
20	(+)-cis-Sabinol	19	14.85	0.13
21	3,5,5-Trimethylcyclohex-3-en-1-one	C20	14.93	0.8
22	Carvenone	C21	15.29	0.39
23	Terpinen-4-ol	C22	15.40	3.39
24	p-Cymen-8-ol	C23	15.49	0.54
25	Cryptone	C24	15.60	5.99
26	cis-Piperitol	C25	15.66	t
27	Sabinyl acetate	C26	15.77	0.42
28	m-Cumenol	C27	15.96	0.41
29	Cuminaldehyde	C28	16.36	4.56
30	Carvotanacetone	C29	16.45	0.14
31	Piperitone	C30	16.55	0.24
32	Limonene dioxide	C31	16.67	t
33	p-Mentha-1,5-dien-7-ol	C32	16.76	0.19
34	Phellandral	C33	16.93	5.34
35	p-Cymen-7-ol	C34	16.93	0.99
36	Thymol	C35	17.03	1.83
37	Camphane	C36	17.65	0.12
38	*α*-Copaene	C37	18.28	0.12
39	Methyl eugenol	C38	18.39	0.12
40	*β*-Elemene	C39	18.44	0.16
41	(−)-*α*-Gurjunene	C40	18.78	0.14
42	*β*-Caryophyllene	C41	18.94	t
43	Viridiflorol	C42	19.02	0.15
44	Aromadendrene	C43	19.20	1.09
45	*α*-Copaen-8-ol	C44	19.38	0.28
46	Aromadendrene, dehydro-	C45	19.43	0.49
47	(−)-Alloaromadendrene	C46	19.50	2.14
48	Isoamyl phenylacetate	C47	19.57	0.43
49	2-Isopropenyl-1,3,5-trimethylbenzene	C48	19.80	0.88
50	Ledene	C49	19.85	0.51
51	Gamma-muurolene	C50	20.05	0.13
52	*β*-Cadinene	C51	20.10	0.13
53	trans-Calamenene	C52	20.14	0.12
54	*α*-Calacorene	C53	20.42	0.12
55	*γ*-Maaliene	C54	20.70	0.48
56	(+)-Spathulenol	C55	20.80	0.40
57	(−)-Spathulenol	C56	20.96	9.42
58	*β*-Humulene	C57	21.03	2.05
59	(+)-*γ*-Gurjunene	C58	21.13	0.21
60	13-Apo-beta-carotenone	C59	21.16	0.32
61	Ledol	C60	21.26	1.1
62	Inconnu	C124	21.56	0.74
63	*γ*-Himachalene	C61	21.73	0.18
64	Epi-Eudesmol	C62	21.79	0.63
65	Inconnu	C125	22.74	0.62
66	Inconnu	C126	22.96	0.24
67	2-Pentadecanone, 6,10,14-trimethyl-	C61	23.41	0.29

	**Monoterpene hydrocarbons**	**MH**		**25.08**
	**Oxygenated monoterpenes**	**OM**		**45.92**
	**Sesquiterpenes hydrocarbons**	**SH**		**8.07**
	**Oxygenated sesquiterpenes**	**OS**		**12.3**
	**Others**	**O**		**5.11**
	**Total**			**96.48**

t: trace. The bold values represent the chemical classes of *Eucalyptus camaldulensis* essential oil and its proportion.

**Table 3:** Chemical composition of *Mentha pulegium* essential oil.

No.	Compound	Abbreviation	RT	Area (%)
1	*α*-Thujene	C1	11.12	t
2	*α*-Pinene	C2	11.30	1.45
3	Artemisiatriene	C64	11.59	0.17
4	*β*-Terpinene	C4	11.99	t
5	*β*-Pinene	C65	12.09	1.08
6	3-Octanol	C66	12.24	2.13
7	*α*-Fenchene	C67	12.52	t
8	O-Cymene	C68	12.85	0.33
9	D-Limonene	C69	12.94	1.87
10	1,8-Cineole	C10	13.02	0.5
11	Inconnu	C127	13.31	0.39
12	p-Mentha-3,8-diene	C70	13.59	0.18
13	3-Octanol acetate	C71	14.29	t
14	Wine lactone	C72	14.34	0.19
15	3,4-Heptadien-2-one,3-cyclopentyl-6-methyl-	C73	14.89	1.32
16	Menthone	C74	15.02	0.73
17	Isopulegol	C75	15.08	t
18	Isomenthone	C76	15.20	0.43
19	Isopulegone	C77	15.38	6.69
20	Inconnu	C128	15.72	0.14
21	*α*-Terpineol	C78	15.83	t
22	Verbenone	C79	16.04	1.00
23	Inconnu	C129	16.24	1.69
24	Pulegone	C80	16.52	51.02
25	D-Isomenthone	C81	16.71	0.94
26	*γ*-Diosphenol	C82	16.80	1.62
27	2-Acetyl-4-methylcyclopentane-1,3-dione	C83	16.85	1.12
28	Thymol	C35	16.93	t
29	(-)-1R-8-Hydroxy-p-menth-4-en-3-one	C84	17.07	4.08
30	Inconnu	C130	17.28	0.72
31	Cyclohexene, 1-acetyl-2-(1-hydroxyethyl)	C85	17.59	0.33
32	Piperitenone	C86	17.86	5.90
34	DL-Camphoric acid	C87	17.95	1.99
35	Cuminol	C88	18.38	0.21
36	Cinerolone	C89	18.62	0.24
37	4,5-Diethyl-3,5-octadiene	C90	19.09	0.88
38	Humulene	C91	19.38	t
39	Isomintlactone	C92	19.95	1.74
10	1,3,3-Trimethyl-2-hydroxymethyl-3,3-dimethyl-4-(3-methylbut-2-enyl)-cyclohexene	C93	20.53	0.84
41	Spathulenol	C55	20.91	t
42	Caryophyllene oxide	C94	21.03	1.53
43	Humulene epoxide II	C95	21.33	2
44	Inconnu	C131	22.60	0.15
45	Phytone	C96	23.41	0.16

	**Monoterpene hydrocarbons**	**MH**		**5.27**
	**Oxygenated monoterpenes**	**OM**		**77.09**
	**Sesquiterpenes hydrocarbons**	**SH**		**t**
	**Oxygenated sesquiterpenes**	**OS**		**3.69**
	**Others**	**O**		**9.72**
	**Total**			**95.77**

t: trace. The bold values represent the chemical classes of *Mentha pulegium* essential oil and its proportion.

**Table 4:** Chemical composition of *Rosmarinus officinalis* essential oil.

No.	Compound	Abbreviation	RT	Area (%)
1	Cyclofenchene	C97	11.12	0.94
2	*α*-Pinene	C2	11.36	24.90
3	3-Carene	C98	11.47	0.45
4	Camphene	C99	11.65	9.23
5	3-Phenylpentan	C100	11.70	1.24
6	1-Octen-3-ol	C101	11.95	0.11
7	*β*-Terpinene	C4	12	0.2
8	*α*-Fenchene	C67	12.12	4.24
9	*β*-Pinene	C65	12.19	2.51
10	*α*-Phellandrene	C7	12.56	0.68
11	*β*-Thujene	C102	12.64	0.24
12	*α*-Terpinolene	C103	12.73	1.68
13	P-Cymene	C9	12.87	2.70
14	D-Limonene	C69	12.97	7.15
15	1.8-Cineole	C10	13.04	1.09
16	*γ*-Terpinene	C12	13.43	3.45
17	4-Thujanol	C104	13.59	0.60
18	4-Carene	C105	13.93	2.59
19	Linalool	C14	14.03	4.3
20	1-Methylene-3-(1-methylethylidene) cyclopentane	C106	14.20	0.56
21	Fenchol	C107	14.42	t
22	Eucarvone	C108	14.57	0.5
23	(S)-cis-Verbenol	C109	14.90	0.3
24	(+)-Camphor	C110	14.99	6.11
25	Pinocarvone	C111	15.21	0.56
26	Borneol	C112	15.27	2.12
27	Terpinen-4-ol	C22	15.39	1.51
28	*α*-Terpineol	C78	15.57	1.04
29	Myrtenol	C113	15.70	0.77
30	Verbenone	C79	15.95	3.51
31	Pulegone	C80	16.33	1.30
32	Methyl (3Z)-3,7-dimethyl-3,6-octadienoate	C114	16.56	0.17
33	Thymol	C35	16.76	0.1
34	Bornyl acetate	C115	16.97	3.57
35	Carvacrol	C116	17.02	0.29
36	Piperitenone	C86	17.79	t
37	*α*-Copaene	C37	18.29	0.16
38	Methyl eugenol	C38	18.39	0.14
39	*β*-Caryophyllene	C41	18.95	2.23
40	Geranylacetone	C117	19.04	0.35
41	Humulene	C91	19.39	1.53
42	*α*-Curcumene	C118	19.50	0.15
43	*γ*-Muurolene	C50	19.56	0.15
44	*α*-Himachalene	C119	19.82	0.82
45	*β*-Cedrene	C120	20.04	0.20
46	*β*-Cadinene	C51	20.10	0.2
47	Caryophyllene oxide	C94	21.02	0.43
48	Humulene epoxide II	C95	21.31	0.19
49	*α*-Elemene	C121	21.55	0.14
50	*α*-Bisabolol	C122	21.91	0.38

	**Monoterpene hydrocarbons**	**MH**		**60.96**
	**Oxygenated monoterpenes**	**OM**		**28.16**
	**Sesquiterpenes hydrocarbons**	**SH**		**5.58**
	**Oxygenated sesquiterpenes**	**OS**		**1**
	**Others**	**O**		**2.08**
	**Total**			**97.78**

t: trace. The bold values represent the chemical classes of *Rosmarinus officinalis* essential oil and its proportion.

**Table 5:** Bacteria inhibition zones of essential oils and antibiotics.

	Inhibition zones (mm)		
	*P. mirabilis*	*K. pneumoniae*	*S. aureus*
Essential oils			
ECEO	25	12	10
MPEO	20	20	10
ROEO	20	10	—

Antibiotics			
E	^*∗*^	R	^*∗*^
OFX	R	S	S
TIC	R	^*∗*^	R
OX	^*∗*^	R	R
AMP	R	R	R
NOR	R	S	S
CAZ	R	R	R
CTX	R	S	R
DMSO	—	—	—

ECEO: *Eucalyptus camaldulensis* essential oil; MPEO: *Mentha pulegium* essential oil; ROEO: *Rosmarinus officinalis* essential oil; E: Erythromycin; OFX: Ofloxacin; TIC: Ticarcillin; OX: Oxacillin; AMP: Ampicillin; NOR: Norfloxacin; CAZ: Ceftazidime; CTX: Cefotaxime. R: resistant; S: sensitive; —: no inhibition. ^*∗*^Antibiotic does not correspond to this strain.

**Table 6:** Minimum inhibitory concentration (MIC) of essential oils ECEO, PMEO, and ROEO against *P. mirabilis*, *K. pneumoniae*, and *S. aureus*.

Concentration % (v:v)	*P. mirabilis*			*K. pneumoniae*			*S. aureus*		
	ECEO	MPEO	ROEO	ECEO	MPEO	ROEO	ECEO	MPEO	ROEO
10	−	−	−	−	−	−	−	−	+
5	−	−	−	−	−	+	−	−	+
2.5	−	−	−	−	−	+	−	−	+
1.25	−	−	+	−	−	+	−	−	+
0.625	−	−	+	−	−	+	−	−	+
0.3125	−	−	+	−	−	+	−	+	+
0.15625	+	+	+	−	−	+	−	+	+
0,078125	+	+	+	−	−	+	−	+	+
0,0390625	+	+	+	−	−	+	+	+	+
0,01953125	+	+	+	+	+	+	+	+	+
0,009765625	+	+	+	+	+	+	+	+	+

ECEO: *Eucalyptus camaldulensis* essential oil; MPEO: *Mentha pulegium* essential oil; ROEO: *Rosmarinus officinalis* essential oil. +: bacterial growth; −: no bacterial growth.

**Table 7:** Different combinations of the essential oil contents chosen by the mixing design and the responses for each bacterial strain.

Experience	Essential oil proportion			Response in MIC_m_ % (v:v)		
	MPEO	ECEO	ROEO	*P. mirabilis*	*K. pneumoniae*	*S. aureus*
1	1.00	0.00	0.00	10	5	10
2	0.00	1.00	0.00	10	10	10
3	0.00	0.00	1.00	5	10	20
4	0.50	0.50	0.00	2.5	10	20
5	0.50	0.00	0.50	10	2.5	20
6	0.00	0.50	0.50	2.5	5	10
7	0.33	0.33	0.33	2.5	5	10
8	0.67	0.17	0.17	2.5	5	10
9	0.17	0.67	0.17	2.5	5	10
10	0.17	0.17	0.67	2.5	2.5	5
11	0.33	0.33	0.33	2.5	5	10
12	0.33	0.33	0.33	2.5	5	10

ECEO: *Eucalyptus camaldulensis* essential oil; MPEO: *Mentha pulegium* essential oil; ROEO: *Rosmarinus officinalis* essential oil; MIC_m_: minimum inhibitory concentration of the mixtures.

**Table 8:** Analysis of variance for models postulated against the studied bacterial strains.

*P. mirabilis*					
Source	Freedom degree	Sum of squares	Mean square	*F* ratio	*p* value
Regression	6	113.223	18.87	9.733	0.0123^*∗*^
Residuals	5	9.693	1.938		
Total	11	122.916			
Lack of fit	3	9.963	3.231		
Pure error	2	0.000	0.000		
*R* ^2^	0.92				
*R*^2^ adjusted	0.83				

*K. pneumoniae*					
Regression	6	69.758	11.626	6.178	0.0321^*∗*^
Residuals	5	9.409	1.881		
Total	11	79.166			
Lack of fit	3	9.409	3.136		
Pure error	2	0.0000	0.000		
*R* ^2^	0.88				
*R*^2^ adjusted	0.74				

*S. aureus*					
Regression	6	201.46	33.577	2.349	0.183
Residuals	5	71.45	14.29		
Total	11	272.917			
Lack of fit	3	71.45	23.818		
Pure error	2	0.000	0.000		
*R* ^2^	0.74				
*R*^2^ adjusted	0.42				

^*∗*^*p* value < 0.05.

**Table 9:** Estimated effects of model coefficients linking responses to factors.

		*P. mirabilis*				*K. pneumoniae*			
Term	Coef	Estimation	SD	Student's *t*-test	*p* value	Estimation	SD	Student's *t*-test	*p* value
MPEO	*b*1	9.40	1.34	6.99	0.0009^*∗*^	5,25	1,32	3,97	0,0107^*∗*^
ECEO	*b*2	10.08	1.34	7.50	0.0007^*∗*^	9,57	1,32	7,23	0,0008^*∗*^
ROEO	*b*3	4.86	1.34	3.61	0.0153^*∗*^	9,57	1,32	7,23	0,00008^*∗*^
MPEO/ECEO	*b*12	−31.02	6.77	−4.58	0.0059^*∗*^	9,65	6,67	1,45	0,2075
MPEO/ROEO	*b*13	8.53	6.77	1.26	0.2635	−20,35	6,67	−3,05	0,0284^*∗*^
ECEO/ROEO	*b*23	−20.11	6.77	−2.97	0.0312^*∗*^	−21,71	6,67	3,25	0,0226^*∗*^
MPEO/ECEO/ROEO	*b*123	−41.4	36.83	−1.12	0.3121	−3,6	36,29	−0,10	0,9248

ECEO: *Eucalyptus camaldulensis* essential oil; MPEO: *Mentha pulegium* essential oil; ROEO: *Rosmarinus officinalis* essential oil; SD: standard deviation; Coef: coefficients. ^*∗*^*p* value < 0.05.

## Data Availability

The datasets used and/or analyzed during the current study are available from the corresponding author upon reasonable request.

## References

[1] Stamm W. E., Norrby S. R. (2001). Urinary tract infections: disease panorama and challenges. *The Journal of Infectious Diseases*.

[2] Rafalskiy V., Pushkar D., Yakovlev S. (2019). Distribution and antibiotic resistance profile of key gram-negative bacteria that cause community-onset urinary tract infections in the Russian Federation: RESOURCE multicentre surveillance 2017 study. *Journal of Global Antimicrobial Resistance*.

[3] Zhu C., Liu H., Wang Y. (2019). Prevalence, incidence, and risk factors of urinary tract infection among immobile inpatients in China: a prospective, multi-centre study. *Journal of Hospital Infection*.

[4] Sheerin N. S., Glover E. K. (2019). Urinary tract infection. *Medicine*.

[5] Bruyere F., Traxer O., Saussine C., Lechevallier E. (2008). Infection et lithiase urinaire. *Progrès en Urologie*.

[6] Puigvert A. (2002). Urinary infection stones. *Urologia Internationalis*.

[7] Rieu P. (2005). Lithiases d’infection. *Annales d’Urologie*.

[8] Pak C. Y. (1998). Kidney stones. *The Lancet*.

[9] Heilberg I. P., Schor N. (2006). Renal stone disease: causes, evaluation and medical treatment. *Arquivos Brasileiros de Endocrinologia & Metabologia*.

[10] Yap P. S., Lim S. H., Hu C. P., Yiap B. C. (2013). Combination of essential oils and antibiotics reduce antibiotic resistance in plasmid-conferred multidrug resistant bacteria. *Phytomedicine: International Journal of Phytotherapy and Phytopharmacology*.

[11] Yap P. S. X., Yiap B. C., Ping H. C., Lim S. H. E. (2014). Essential oils, a new horizon in combating bacterial antibiotic resistance. *The Open Microbiology Journal*.

[12] Ouedrhiri W., Balouiri M., Bouhdid S. (2016). Mixture design of *Origanum compactum*, *Origanum majorana* and *Thymus serpyllum* essential oils: optimization of their antibacterial effect. *Industrial Crops and Products*.

[13] Fadil M., Fikri-Benbrahim K., Rachiq S. (2018). Combined treatment of *Thymus vulgaris L*., *Rosmarinus officinalis L*. and *Myrtus communis L*. essential oils against *Salmonella typhimurium*: optimization of antibacterial activity by mixture design methodology. *European Journal of Pharmaceutics and Biopharmaceutics*.

[14] Blonk B., Cock I. E. (2019). Interactive antimicrobial and toxicity profiles of Pittosporum angustifolium Lodd. extracts with conventional antimicrobials. *Journal of Integrative Medicine*.

[15] Elaissi A., Rouis Z., Salem N. A. B. (2012). Chemical composition of 8 eucalyptus species’ essential oils and the evaluation of their antibacterial, antifungal and antiviral activities. *BMC Complementary and Alternative Medicine*.

[16] Batish D. R., Singh H. P., Kohli R. K., Kaur S. (2008). Eucalyptus essential oil as a natural pesticide. *Forest Ecology and Management*.

[17] Haouel S., Mediouni-Ben Jemâa J., Khouja M. (2010). Postharvest control of the date moth ectomyelois ceratoniae using eucalyptus essential oil fumigation. *Tunisian Journal of Plant Protection*.

[18] Medhi S. M., Reza S., Mahnaz K. (2010). Phytochemistry and larvicidal activity of *Eucalyptus camaldulensis* against malaria vector, *Anopheles stephensi*. *Asian Pacific Journal of Tropical Medicine*.

[19] Takahashi T., Kokubo R., Sakaino M. (2004). Antimicrobial activities of eucalyptus leaf extracts and flavonoids from Eucalyptus maculata. *Letters in Applied Microbiology*.

[20] Boulekbache-Makhlouf L., Meudec E., Chibane M. (2010). Analysis by high-performance liquid chromatography diode array detection mass spectrometry of phenolic compounds in fruit of Eucalyptus globulus cultivated in Algeria. *Journal of Agricultural and Food Chemistry*.

[21] Boulekbache-Makhlouf L., Slimani S., Madani K. (2013). Total phenolic content, antioxidant and antibacterial activities of fruits of Eucalyptus globulus cultivated in Algeria. *Industrial Crops and Products*.

[22] Chinnarasu C., Montes A., Fernandez-Ponce M. T. (2015). Natural antioxidant fine particles recovery from Eucalyptus globulus leaves using supercritical carbon dioxide assisted processes. *The Journal of Supercritical Fluids*.

[23] Harkat-Madouri L., Asma B., Madani K. (2015). Chemical composition, antibacterial and antioxidant activities of essential oil of Eucalyptus globulus from Algeria. *Industrial Crops and Products*.

[24] Luís Â., Duarte A., Gominho J., Domingues F., Duarte A. P. (2016). Chemical composition, antioxidant, antibacterial and anti-quorum sensing activities of Eucalyptus globulus and Eucalyptus radiata essential oils. *Industrial Crops and Products*.

[25] Safaei-Ghomi J., Ahd A. (2010). Antimicrobial and antifungal properties of the essential oil and methanol extracts of *Eucalyptus largiflorens* and Eucalyptus intertexta. *Pharmacognosy Magazine*.

[26] Barbosa L., Filomeno C., Teixeira R. (2016). Chemical variability and biological activities of Eucalyptus spp. essential oils. *Molecules*.

[27] Salehi B., Sharifi-Rad J., Quispe C. (2019). Insights into Eucalyptus genus chemical constituents, biological activities and health-promoting effects. *Trends in Food Science & Technology*.

[28] Teixeira B., Marques A., Ramos C. (2012). European pennyroyal (Mentha pulegium) from Portugal: chemical composition of essential oil and antioxidant and antimicrobial properties of extracts and essential oil. *Industrial Crops and Products*.

[29] Ouakouak H., Chohra M., Denane M. (2015). Chemical composition, antioxidant activities of the essential oil of *Mentha pulegium L*, South East of Algeria. *International Letters of Natural Sciences*.

[30] Baali F., Boumerfeg S., Napoli E. (2019). Chemical composition and biological activities of essential oils from two wild Algerian medicinal plants: *Mentha pulegium L*. and *Lavandula stoechas L*.. *Journal of Essential Oil Bearing Plants*.

[31] Jain S., Jain D. K., Balekar N. (2012). In-vivo antioxidant activity of ethanolic extract of Mentha pulegium leaf against CCl4 induced toxicity in rats. *Asian Pacific Journal of Tropical Biomedicine*.

[32] M. Domingues P., Santos L. (2019). Essential oil of pennyroyal (Mentha pulegium): composition and applications as alternatives to pesticides-new tendencies. *Industrial Crops and Products*.

[33] Mahboubi M., Haghi G. (2008). Antimicrobial activity and chemical composition of *Mentha pulegium L*. essential oil. *Journal of Ethnopharmacology*.

[34] Ahmed A., Ayoub K., Chaima A. J., Hanaa L., Abdelaziz C. (2018). Effect of drying methods on yield, chemical composition and bioactivities of essential oil obtained from Moroccan *Mentha pulegium L*. *Biocatalysis and Agricultural Biotechnology*.

[35] Ekrami M., Emam-Djomeh Z., Ghoreishy S. A., Najari Z., Shakoury N. (2019). Characterization of a high-performance edible film based on Salep mucilage functionalized with pennyroyal (Mentha pulegium). *International Journal of Biological Macromolecules*.

[36] Moore J., Yousef M., Tsiani E. (2016). Anticancer effects of rosemary (*Rosmarinus officinalis L*.) extract and rosemary extract polyphenols. *Nutrients*.

[37] Pereira P. S., Maia A. J., Tintino S. R. (2017). Trypanocide, antileishmania and cytotoxic activities of the essential oil from *Rosmarinus officinalis L* in vitro. *Industrial Crops and Products*.

[38] Zoral M. A., Futami K., Endo M., Maita M., Katagiri T. (2017). Anthelmintic activity of Rosmarinus officinalis against Dactylogyrus minutus (Monogenea) infections in *Cyprinus carpio*. *Veterinary Parasitology*.

[39] Wang W., Wu N., Zu Y. G., Fu Y. J. (2008). Antioxidative activity of *Rosmarinus officinalis L*. essential oil compared to its main components. *Food Chemistry*.

[40] Ghasemzadeh M. R., Amin B., Mehri S., Mirnajafi-Zadeh S. J., Hosseinzadeh H. (2016). Effect of alcoholic extract of aerial parts of *Rosmarinus officinalis L*. on pain, inflammation and apoptosis induced by chronic constriction injury (CCI) model of neuropathic pain in rats. *Journal of Ethnopharmacology*.

[41] Habtemariam S. (2016). The therapeutic potential of rosemary (Rosmarinus officinalis) diterpenes for alzheimer’s disease. *Evidence-Based Complementary and Alternative Medicine*.

[42] Ali A., Chua B. L., Chow Y. H. (2019). An insight into the extraction and fractionation technologies of the essential oils and bioactive compounds in *Rosmarinus officinalis L*.: past, present and future. *TrAC Trends in Analytical Chemistry*.

[43] Bilska A., Kobus-Cisowska J., Kmiecik D. (2019). Cholinesterase inhibitory activity, antioxidative potential and microbial stability of innovative liver pâté fortified with rosemary extract (Rosmarinus officinalis). *Electronic Journal of Biotechnology*.

[44] Karadağ A. E., Demirci B., Çaşkurlu A. (2019). In vitro antibacterial, antioxidant, anti-inflammatory and analgesic evaluation of *Rosmarinus officinalis L*. flower extract fractions. *South African Journal of Botany*.

[45] Pintore G., Usai M., Bradesi P. (2002). Chemical composition and antimicrobial activity of *Rosmarinus officinalis L*. oils from Sardinia and Corsica. *Flavour and Fragrance Journal*.

[46] Del Baño M. J., Lorente J., Castillo J. (2004). Flavonoid distribution during the development of leaves, flowers, stems, and roots of Rosmarinus officinalis. Postulation of a biosynthetic pathway. *Journal of Agricultural and Food Chemistry*.

[47] Bozin B., Mimica-Dukic N., Samojlik I., Jovin E. (2007). Antimicrobial and antioxidant properties of Rosemary and Sage (*Rosmarinus officinalis L*. and *Salvia officinalis L*., Lamiaceae) essential oils. *Journal of Agricultural and Food Chemistry*.

[48] Bai N., He K., Roller M. (2010). Flavonoids and phenolic compounds from Rosmarinus officinalis. *Journal of Agricultural and Food Chemistry*.

[49] Lou Z., Chen J., Yu F. (2017). The antioxidant, antibacterial, antibiofilm activity of essential oil from *Citrus medica L*. var. sarcodactylis and its nanoemulsion. *LWT*.

[50] EUCAST

[51] Abdelli M., Moghrani H., Aboun A., Maachi R. (2016). Algerian *Mentha pulegium L*. leaves essential oil: chemical composition, antimicrobial, insecticidal and antioxidant activities. *Industrial Crops and Products*.

[52] Sadiki M., Balouiri M., Barkai H. (2014). Synergistic antibacterial effect of Myrtus communis and Thymus vulgaris essential oils fractional inhibitory concentration index. *International Journal of Pharmacy and Pharmaceutical Sciences*.

[53] Goupy J., Creighton L. (2006). *Introduction Aux Plans D’expériences*.

[54] Mandal N. K., Pal M., Aggarwal M. L. (2012). Pseudo-Bayesian-optimal designs for estimating the point of maximum in component-amount Darroch-Waller mixture model. *Statistics & Probability Letters*.

[55] Varanda C., Portugal I., Ribeiro J., Silva A. M. S., Silva C. M. (2017). Optimization of bitumen formulations using mixture design of experiments (MDOE). *Construction and Building Materials*.

[56] Elgat W. A. A. A., Kordy A. M., Böhm M., Černý R., Abdel-Megeed A., Salem M. Z. M. (2020). *Eucalyptus camaldulensis*, citrus aurantium, and citrus sinensis essential oils as antifungal activity against Aspergillus flavus, Aspergillus Niger, Aspergillus terreus, and Fusarium culmorum. *Processes*.

[57] Knezevic P., Aleksic V., Simin N., Svircev E., Petrovic A., Mimica-Dukic N. (2016). Antimicrobial activity of *Eucalyptus camaldulensis* essential oils and their interactions with conventional antimicrobial agents against multi-drug resistant Acinetobacter baumannii. *Journal of Ethnopharmacology*.

[58] Farah A., Satrani B., Fechtal M., Chaouch A., Talbi M. (2001). Composition chimique et activités antibactérienne et antifongique des huiles essentielles extraites des feuilles d’Eucalyptus camaldulensiset de son hybride naturel (clone 583). *Acta Botanica Gallica*.

[59] Farah A., Fechtal M., Chaouch A. (2002). Effet du sens du croisement sur la teneur et la composition chimique des huiles essentielles des différents hybrides d’Eucalyptus cultivés au Maroc. *Annals of Forest Science*.

[60] Brahmi F., Abdenour A., Bruno M. (2016). Chemical composition and in vitro antimicrobial, insecticidal and antioxidant activities of the essential oils of *Mentha pulegium L*. and *Mentha rotundifolia* (L.) Huds growing in Algeria. *Industrial Crops and Products*.

[61] Bouyahya A., Et-Touys A., Bakri Y. (2017). Chemical composition of Mentha pulegium and Rosmarinus officinalis essential oils and their antileishmanial, antibacterial and antioxidant activities. *Microbial Pathogenesis*.

[62] Chraibi M., Farah A., Lebrazi S., El Amine O., Iraqui Houssaini M., Fikri-Benbrahim K. (2016). Antimycobacterial natural products from Moroccan medicinal plants: chemical composition, bacteriostatic and bactericidal profile of Thymus satureioides and Mentha pulegium essential oils. *Asian Pacific Journal of Tropical Biomedicine*.

[63] Liu T., Sui X., Zhang R. (2011). Application of ionic liquids based microwave-assisted simultaneous extraction of carnosic acid, rosmarinic acid and essential oil from Rosmarinus officinalis. *Journal of Chromatography A*.

[64] Ainane A., Khammour F., Charaf S. (2019). Chemical composition and insecticidal activity of five essential oils: cedrus atlantica, Citrus limonum, Rosmarinus officinalis, Syzygium aromaticum and Eucalyptus globules. *Materials Today: Proceedings*.

[65] Capatina L., Boiangiu R. S., Dumitru G. (2020). Rosmarinus officinalis essential oil improves scopolamine-induced neurobehavioral changes via restoration of cholinergic function and brain antioxidant status in zebrafish (*Danio rerio*). *Antioxidants*.

[66] Barreto H. M., Silva Filho E. C., Lima E. D. O. (2014). Chemical composition and possible use as adjuvant of the antibiotic therapy of the essential oil of Rosmarinus officinalis L. *Industrial Crops and Products*.

[67] Selmi S., Rtibi K., Grami D., Sebai H., Marzouki L. (2017). Rosemary (Rosmarinus officinalis) essential oil components exhibit anti-hyperglycemic, anti-hyperlipidemic and antioxidant effects in experimental diabetes. *Pathophysiology*.

[68] Mattazi N., Farah A., Fadil M., Chraibi M., Benbrahim K. F. (2015). Essential oils analysis and antibacterial activity of the leaves of Rosmarinus officinalis, Salvia officinalis and Mentha piperita cultivated in agadir (Morocco). *International Journal of Pharmacy and Pharmaceutical Sciences*.

[69] Ložienė K., Švedienė J., Paškevičius A. (2018). Influence of plant origin natural *α*-pinene with different enantiomeric composition on bacteria, yeasts and fungi. *Fitoterapia*.

[70] Duru M. E., Öztürk M., Uğur A., Ceylan Ö. (2004). The constituents of essential oil and in vitro antimicrobial activity of Micromeria cilicica from Turkey. *Journal of Ethnopharmacology*.

[71] Santoyo S., Cavero S., Jaime L., Ibañez E., Señoráns F. J., Reglero G. (2005). Chemical composition and antimicrobial activity of *Rosmarinus officinalis L.* essential oil obtained via supercritical fluid extraction. *Journal of Food Protection*.

[72] Hussain A. I., Anwar F., Nigam P. S., Ashraf M., Gilani A. H. (2010). Seasonal variation in content, chemical composition and antimicrobial and cytotoxic activities of essential oils from four Mentha species. *Journal of the Science of Food and Agriculture*.

[73] Van Vuuren S. F., Viljoen A. M. (2007). Antimicrobial activity of limonene enantiomers and 1,8-cineole alone and in combination. *Flavour and Fragrance Journal*.

[74] Shahverdi A., Rafii F., Fazeli M. R., Jamalifar H. (2004). Enhancement of antimicrobial activity of furazolidone and nitrofurantoin against clinical isolates of Enterobacteriaceae by piperitone. *International Journal of Aromatherapy*.

[75] Tiwari B. K., Valdramidis V. P., O’ Donnell C. P., Muthukumarappan K., Bourke P., Cullen P. J. (2009). Application of natural antimicrobials for food preservation. *Journal of Agricultural and Food Chemistry*.

[76] Bouyahya A., Bakri Y., Et-Touys A. (2017). Résistance aux antibiotiques et mécanismes d’action des huiles essentielles contre les bactéries. *Phytothérapie*.

[77] Xiang F., Bai J., Tan X., Chen T., Yang W., He F. (2018). Antimicrobial activities and mechanism of the essential oil from *Artemisia argyi* Levl. et Van. var. argyi cv. Qiai. *Industrial Crops and Products*.

